# Challenges in Diversity, Equity, and Inclusion in Research and Clinical Oncology

**DOI:** 10.3389/fonc.2021.642112

**Published:** 2021-03-24

**Authors:** Wafik S. El-Deiry, Giuseppe Giaccone

**Affiliations:** ^1^ Cancer Center, Brown University, Providence, RI, United States; ^2^ Weill Cornell Medical College, New York, NY, United States

**Keywords:** diversity, inclusion, oncology, patient outcomes, clinical research, disparities

## Abstract

Disparities are common and well-known in the field of clinical oncology and cancer research. In patient care, poor access and a number of other factors disadvantage patients and this can lead to inadequate screening, prevention or treatment of cancer and poor patient outcomes. World-wide, socioeconomic status, health care expenditures and a number of other challenges contribute to disparities in cancer care and patient outcomes. Access to cancer clinical trials remains inadequate for underrepresented minorities as well as non-white racial and ethnic groups. There are also disparities and many challenges in the biomedical research enterprise that can limit innovation and that must be addressed as part of active interventions.

## Disparities in Cancer Care

There is much evidence in support of disparities as an important factor in patient outcomes in the field of oncology (1–4), and this has become even more apparent in the COVID era (5, 6). Curable cancers are not screened as they should be, only to be diagnosed at an advanced stage which is more difficult to treat and which is associated with poor patient survival. The factors leading to these disparities range from lack of education and outreach to poor individuals in underserved communities, coupled with less access and lack of affordability of care (7, 8). While everyone faces the issues of increasingly more expensive healthcare and drug costs, the quality of insurance coverage including secondary coverage impacts on the care that is provided as well as the ability of poor and underserved patients to take part of it (9).

## World-Wide, Socioeconomic Status, Health Care Expenditures and Other Challenges Contribute to Disparities in Cancer Care and Patient Outcomes

Socioeconomic status is an important contributing factor in the quality of cancer care and disparities in patient survival. For example, in the UK which has differences in survival among patients with colorectal cancer as a function of socioeconomic status, a recent study of nearly 70,000 patients diagnosed with colon cancer between 2010-2013 identified a 21% emergency presentation rate among the affluent and a higher emergency presentation rate of 28% among the most socioeconomically-deprived (10). The differences in emergency presentation were associated with a greater percentage of socioeconomically-deprived patients requiring emergency surgery as compared to the more affluent group (10). The authors concluded that reduced emergency presentations and the need for urgent surgery should be policy targets (10).

Health care expenditures as a function of gross domestic product can contribute to differences in patient outcomes. For example, the availability of imaging technologies, which can be a consequence of per capita health care expenditures as a percentage of gross domestic product, has been associated with favorable mortality-to-incidence ratios in kidney cancer in an analysis from 56 countries (11). In the same tumor type, there are some more expensive modern therapies such as VEGF inhibitors or immunotherapy that provide patients with advanced disease with potential for improved survival beyond surgery alone (11).

Among patients with advanced breast cancer there are a number of challenges that contribute to disparities in outcomes among underserved patient populations. Such disparities have been classified at the level of the individual or at a healthcare system level in a recent pan-European study that convened an expert panel (12). A number of challenges faced by underserved patient populations were identified including awareness, issues with communication, cultural factors, issues with data collection and clinical trial participation, issues with implementation of high-quality guidelines, and some workplace issues (12). Coordinated efforts, including cooperation between countries, to address the challenges that lead to healthcare disparities among patients with metastatic breast cancer could improve outcomes and reduce disparities among the underserved patient populations (12).

## Community Outreach and Impact on Cancer Outcomes

In the United States, NCI-designated cancer centers are committed to programs in community outreach for different racial and ethnic groups and educational programs (13). These efforts can address some barriers in communication and can facilitate altered behaviors that may impact on cancer screening and prevention efforts. It has been estimated by the American Cancer Society that 50% of cancers could be eliminated through lifestyle and behavior modifications or vaccination programs and this of course is low hanging fruit in the world of clinical oncology as cancer prevention is a much easier way to deal with cancer than having to treat advanced disease (14, 15).

## Disparities in Cancer Clinical Trials

But access to care and affordability are only part of the problem with health care disparities and inequities in oncology. It is clear that currently all the major interventions in prevention and therapeutic advances occur through testing in clinical trials. Clinical trials are part of the process that ultimately allows FDA approval of drugs, devices, and population interventions such as vaccinations. It is well-documented that minority populations and often non-white ethnic groups are much less represented in clinical trials (16, 17). Although large clinical trials sponsored by the pharmaceutical industry have become global, inclusion of minorities (e.g. blacks) remains very limited. Some of the issues are related to and may be addressed by outreach but other barriers to inclusion derive from cultural and historical trust issues including “human experimentation” involving certain communities or vulnerable populations (18).

It is only with community outreach and education and work within the community that cancer centers can hope to impact on the barriers to clinical trial enrollment and inclusivity. Erosion of trust in the medical system due to historical victimization of groups or individuals in human experimentation has impacted on the willingness of racial and ethnic minorities to participate in clinical trials (19). Acknowledgments of historical mistakes is a step towards impacting on how clinical trials are conducted, and in some cases the acknowledgements may need to be personal. A good example includes tributes to Henrietta Lacks and her family for their contributions to medical science (20, 21). But there are other obstacles and challenges in the enrollment of racial and ethnic minorities in clinical trials. Such obstacles include access to care, education and communication gaps (19). Many interventions that are part of clinical trials include standard of care such as approved drugs which if not covered by insurance (or if the individuals have no insurance coverage) can add to the barriers and challenges that must be overcome to improve the inclusion of minorities and underserved racial and ethnic groups (22, 23).

## Limitations in Basic Science Widens Disparities

Throughout the world, the laboratory discoveries that come about from basic research are foundational as far as progress that can in the future impact on patient care. It has become clear that lack of attention to minority populations and various racial and ethnic groups has led to a knowledge gap in our understanding of cancer. The largest genomics database known as “The Cancer Genome Atlas” or TCGA has little information on minorities or different racial and ethnic groups (24, 25). It is known however that the severity of the disease can vary in different populations and that there are genetic polymorphisms that may explain the underlying differences. Cancer suppressor genes such as p53 have variants in different populations that impact on its function and its ability to suppress cancer (26, 27). Other examples include cancer susceptibility genes such as BRCA1 and BRCA2 that occur more commonly in Ashkenazi Jewish populations (28).

In recent years there has been growing emphasis on scientific studies on biological variables such as gender and minorities including racial and ethnic groups (29, 30). While there is some improvement in clinical trial enrollment, there remains a major gap and much progress to be made. Attention to these issues will improve our ability to understand cancer biology in different contexts from the biological behavior of cancer in different groups to the metabolism and toxicity of drugs in different hosts to the efficacy of the therapeutics. Indeed, there is a need to address diversity and equity if we are to fulfill the promise of precision medicine to provide the best care possible to each individual (31–33).

## Disparities in Access to the Published Literature

As basic scientists and clinicians undertake efforts to perform basic and clinical research or clinical care in oncology there is a world-wide challenge often faced with lack of access to primary literature (34). This includes historic papers in major journals that remain behind a paywall inaccessible to anyone. This may even include manuscripts investigators authored themselves and paid to publish but cannot access as their own publications. This situation extends to students, and members of the public whose taxes often supported the research. It also extends to populations of other countries where access to the literature is limited or restricted. In the old world of a few decades ago, individuals would go to the library and access the literature for free. In today’s online world this is no longer possible in many cases. Efforts have been mobilized to ensure that NIH funded research is accessible, however often with delays of a year or more. While some journals are allowing immediate open access for a fee to authors, the problem still remains with large amounts of inaccessible especially older literature that should be freely available.

## Lack of Data Sharing Limits Progress in the Field of Cancer Research

The creative process that leads to scientific discoveries and therapeutic advances to help reduce the burden of cancer needs all the help it can get. Information flow and data sharing are high priority areas for improvement. One of the great contributions of the Biden Cancer Moonshot in the United States was a recognition that breaking down silos and allowing free exchange of knowledge could accelerate life-saving discoveries (35, 36). Access to data is like access to the literature. Having more experts look at problems from different points of view is a key to advancing knowledge. This is also true in research laboratories where the greater the diversity of members the higher the chance that certain breakthroughs may be achieved.

## Inequities in Biomedical Research

While we address the inequities described, in our opinion, we need to pay attention to the viability of the biomedical research enterprise. There are huge inequalities from the elite well-funded laboratories at elite institutions, the enormous endowments and prestigious foundations that support them, to the “soldiers” in the field trying to do research against all odds with NIH grant pay-lines in single digits. These inequalities have in the last two decades created a culture of “haves” and “have-nots” with widening gaps as technologies advance and institutions build up their research infrastructure. These realities of how research is conducted in the US, in terms of available resources, are threatening the future of biomedical research as a career in research may no longer be appealing to the brightest students.

There is a grave danger, in our opinion, in the lack of support for science and for the investigators who pursue it. These very investigators have had to deal with ever increasing regulatory burdens in laboratory, animal research or human subjects research as well and the burdens of grant writing that are seemingly never ending. It really doesn’t make sense nor is it acceptable in any profession for researchers who are hired as faculty based on their outstanding accomplishments to be for the rest of their career trying to secure significant portions of their salaries on very competitive grants.

The situation is even more challenging for those who also practice medicine and have to deal with some of the very challenges mentioned earlier such as communication with insurance companies to approve needed care for patients. There is also the ever-increasing burden of clinical documentation with electronic health records that hinder the physician-patient relationship, rather than helping it.

Efforts to enhance diversity and inclusion in the work-force need to consider these types of challenges that are faced and which get in the way of needed progress. Solutions must address not only these issues but obstacles faced by young families and women faculty who often juggle their academic careers with child care and care of family members such as elderly parents. Solutions at our great institutions must include having role models and mentors and an environment that is cognizant of the challenges and is actively working to deal with them. For example Cancer Research UK (CRUK) has various support networks (https://www.cancerresearchuk.org/about-us/charity-jobs/working-with-us/equality-diversity-and-inclusion) and flexible mechanisms to support research activities (https://www.cancerresearchuk.org/funding-for-researchers/applying-for-funding/policies-that-affect-your-grant/flexible-research-careers-funding-policies?_gl=1*7mecl7*_gaMTIwNDE3MTQ5Ny4xNjE0ODE3NDI*_ga_58736Z2GNN*MTYxNDgxNzQyMC4xLjEuMTYxNDgxNzcyNS4yNQ.&_ga=2.168422753.1163472191.1614817421-1204171497.1614817421). Efforts have begun to educate faculty at most US medical schools about conscious and unconscious biases that have relevance to everything that goes on in academia, and which present barriers and widen the gaps in disparities. Early efforts through the NIH are beginning to address some of these issues to facilitate greater minority recruitment of biomedical faculty at US Universities (https://www.nih.gov/news-events/news-releases/nih-fund-cohort-recruitment-development-program-enhance-diversity-inclusion-among-biomedical-faculty).

## Author Contributions

All authors contributed to the article and approved the submitted version.

## Conflict of Interest

The authors declare that the research was conducted in the absence of any commercial or financial relationships that could be construed as a potential conflict of interest.
